# Corrigendum: Metabolic-imaging of human glioblastoma live tumors: a new precision-medicine approach to predict tumor treatment response early

**DOI:** 10.3389/fonc.2025.1588290

**Published:** 2025-04-24

**Authors:** Mariangela Morelli, Francesca Lessi, Serena Barachini, Romano Liotti, Nicola Montemurro, Paolo Perrini, Orazio Santo Santonocito, Carlo Gambacciani, Matija Snuderl, Francesco Pieri, Filippo Aquila, Azzurra Farnesi, Antonio Giuseppe Naccarato, Paolo Viacava, Francesco Cardarelli, Gianmarco Ferri, Paul Mulholland, Diego Ottaviani, Fabiola Paiar, Gaetano Liberti, Francesco Pasqualetti, Michele Menicagli, Paolo Aretini, Giovanni Signore, Sara Franceschi, Chiara Maria Mazzanti

**Affiliations:** ^1^ Section of Genomics and Transcriptomics, Fondazione Pisana per la Scienza, San Giuliano Terme, Pisa, Italy; ^2^ Department of Clinical and Experimental Medicine, University of Pisa, Pisa, Italy; ^3^ Department of Biology, University of Pisa, Pisa, Italy; ^4^ Department of Neurosurgery, Azienda Ospedaliera Universitaria Pisana, Pisa, Italy; ^5^ Neurosurgical Department of Spedali Riuniti di Livorno, Livorno, Italy; ^6^ Department of Pathology, New York University (NYU) Langone Medical Center, New York City, NY, United States; ^7^ Department of Translational Research and New Technologies in Medicine and Surgery, University of Pisa, Pisa, Italy; ^8^ Anatomical Pathology Department, Azienda Ospedaliera Toscana Nord-ovest, Livorno, Italy; ^9^ National Enterprise for nanoScience and nanoTechnology (NEST), Scuola Normale Superiore and Istituto Nanoscienze-CNR, Pisa, Italy; ^10^ Section of Nanomedicine, Fondazione Pisana per la Scienza, San Giuliano Terme, Pisa, Italy; ^11^ Department of Oncology, University College London Hospitals, London, United Kingdom; ^12^ Department of Radiation Oncology, Azienda Ospedaliera Universitaria Pisana, University of Pisa, Pisa, Italy; ^13^ Department of Oncology, University of Oxford, Oxford, United Kingdom; ^14^ Section of Bioinformatics, Fondazione Pisana per la Scienza, San Giuliano Terme, Pisa, Italy

**Keywords:** glioblastoma, metabolic imaging, drug response assay, predictive model, FLIM (fluorescence lifetime imaging microscopy)

In the published article, there was an error in **Figure 1** as published. The two figures above and below in **Figures 1K-L**, related to SOX2 staining, are part of a series of photographs aimed at demonstrating that GB explants—small tissue fragments approximately 300 µm in size—retain the original cytoarchitecture of the tissue from which they are derived (surgery tissue). The upper figure represents an image of the surgery tissue, while the lower figure shows the corresponding small explant (GB-EXP) derived from it. The SOX2 staining was specifically performed to add further evidence of the preserved representation of tumor components within the GB explant, a conclusion also supported by other images in the series (GFAP; CD105,CD33).

**Figure 1:**
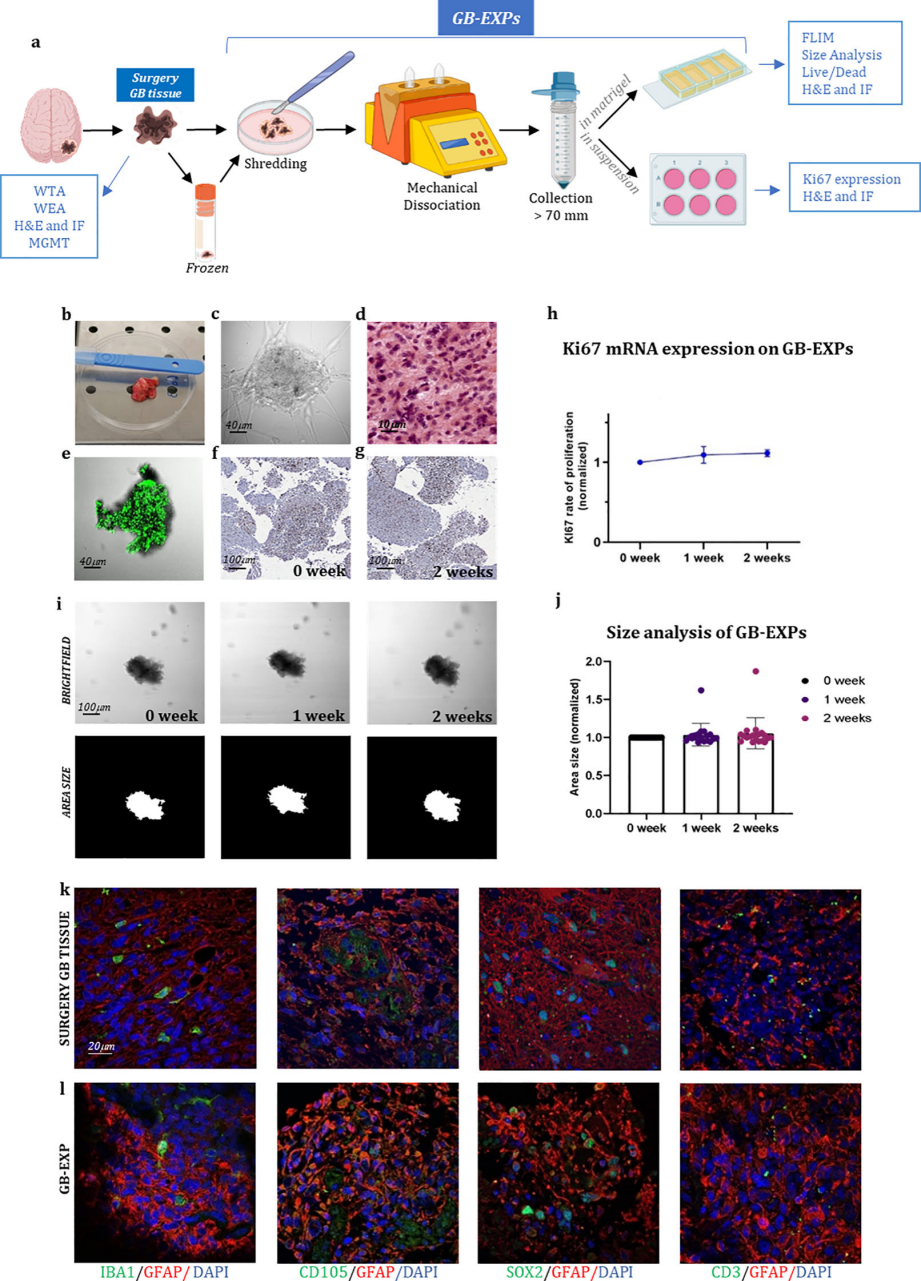
GB-EXPs cultures **(A)** Experimental design. **(B)** A surgery tumor sample. **(C)** GB-EXP embedded in matrigel. **(D)** H&E staining of a surgery GB tissue. **(E)** Live/dead staining of a GB-EXP at 2 weeks after culturing. **(F, G)** Ki67 immunostaining of GB-EXPs in suspension at 0 week and 2 weeks. **(H)** Ki67 mRNA expression analysis of 13 GB case-derived GB-EXPs at 0 and 2 weeks. Graph represents mean ± s.d. of triplicated measures. **(I)** Representative brightfield and area size images of a GB-EXP in matrigel at 0 week, 1 week and 2 weeks. **(J)** Size analysis 20 GB case-derived GB-EXPs at 0, 1 and 2 weeks. **(K, L)** Immunofluorescence assays of surgery GB tissue **(K)** and GB-EXPs **(L)**. GFAP (astrocytes), IBA1 (microglia), SOX2 (stem cells), CD105 (endothelial cells) and CD3 (lymphocytes). WTA, Whole Transcriptome Analysis; WEA, Whole Exome Analysis; FC, Flow Cytometry; H&E, Hematoxylin, and Eosin; MGMT, MGMT promoter methylation analysis.

We acknowledge an error of duplication in the figures related to SOX2, as the upper image is, in fact, a photo of the same explant but with a slightly shifted area. We deeply regret this misplacement and are providing the correct image of the tumor stained with SOX2.The corrected **Figure 1** and its legend appear below.

The authors apologize for this error and state that this does not change the scientific conclusions of the article in any way. The original article has been updated.

